# Diagnosis and clinical management of canine leishmaniosis by general veterinary practitioners: a questionnaire-based survey in Portugal

**DOI:** 10.1186/s13071-021-04799-y

**Published:** 2021-06-07

**Authors:** Marta Monteiro, Sara Prata, Luís Cardoso, Isabel Pereira da Fonseca, Rodolfo Oliveira Leal

**Affiliations:** 1 Hospital Escolar Veterinário, Faculdade de Medicina Veterinária, Universidade de Lisboa, Lisbon, Portugal; 2 Department of Veterinary Sciences, and Animal and Veterinary Research Centre (CECAV), University of Trás-Os-Montes e Alto Douro (UTAD), Vila Real, Portugal; 3 Centro de Investigação Interdisciplinar em Sanidade Animal (CIISA), Faculdade de Medicina Veterinária, Universidade de Lisboa, Lisbon, Portugal

**Keywords:** Antileishmanial drugs, Canine leishmaniosis, Prevention, Questionnaire-based survey

## Abstract

**Background:**

Canine leishmaniosis (CanL) can be appropriately managed following international recommendations. However, few studies have assessed the preferred protocols in real-life veterinary practice and whether these are in line with the guidelines. This survey aimed to investigate the current trends in the clinical management of CanL among veterinary practitioners in Portugal, taking into consideration different scenarios of infection/disease and the awareness of and application by veterinary practitioners of the current guidelines.

**Methods:**

A questionnaire-based survey was conducted online using an electronic platform. The following topics were surveyed: (i) general characteristics of the responding veterinarian; (ii) the preferred protocols used for the diagnosis, treatment and prevention of CanL, considering different theoretical scenarios of infection/disease; and (iii) the responding veterinarian’s current knowledge and application of the existing guidelines on CanL. After internal validation, the survey was distributed online, for 2 months,* via* Portuguese social network veterinary groups. Data were collected for descriptive analysis.

**Results:**

Eighty-six replies were obtained. Analysis of the results showed that the preferred diagnostic techniques varied widely according to the theoretical scenario of infection/disease. In general daily practice, serology testing (enzyme-linked immunosorbent assay [ELISA]) was the most used tool (67.4%). The preferred matrices used for PCR test were lymph nodes (62.3%) and/or bone marrow (59.0%). Regarding treatment, for subclinical infection/stage I CanL, 51.2% of the respondents did not prescribe any medical treatment, but 98.8% proceeded with both monitoring and preventive measures. Among those who prescribed a treatment (*n* = 42), most chose domperidone (47.6%). For the treament of stages IIa, IIb and III CanL, allopurinol/meglumine antimoniate (MA) was chosen by 69.8, 73.3 and 51.2% of respondents, respectively, followed by allopurinol/miltefosine (20.9, 19.8 and 38.4%, respectively). In contrast, dogs with stage IV CanL were mostly treated with allopurinol/miltefosine (48.8%) rather than with allopurinol/MA (23.3%). The use of repellents was the preferred preventive strategy (98.8%). About 93.0% of responders were aware of the existence of guidelines, and most of these veterinarians consulted the guidelines of the LeishVet group and the Canine Leishmaniosis Working Group; however, 31.3% reported that they did not follow any specific recommendations.

**Conclusions:**

Of the veterinarians responding to the survey, most reported following international guidelines for the clinical management of CanL. While allopurinol/MA was the preferred therapeutic protocol for the treatment of stages II/III CanL, allopurinol/miltefosine was the first choice for the treatment of stage IV CanL, possibly due to the unpredictable effect of MA on renal function. This study contributes to a better understanding of the trends in practical approaches to the treatment of CanL in Portugal.

**Graphical Abstract:**

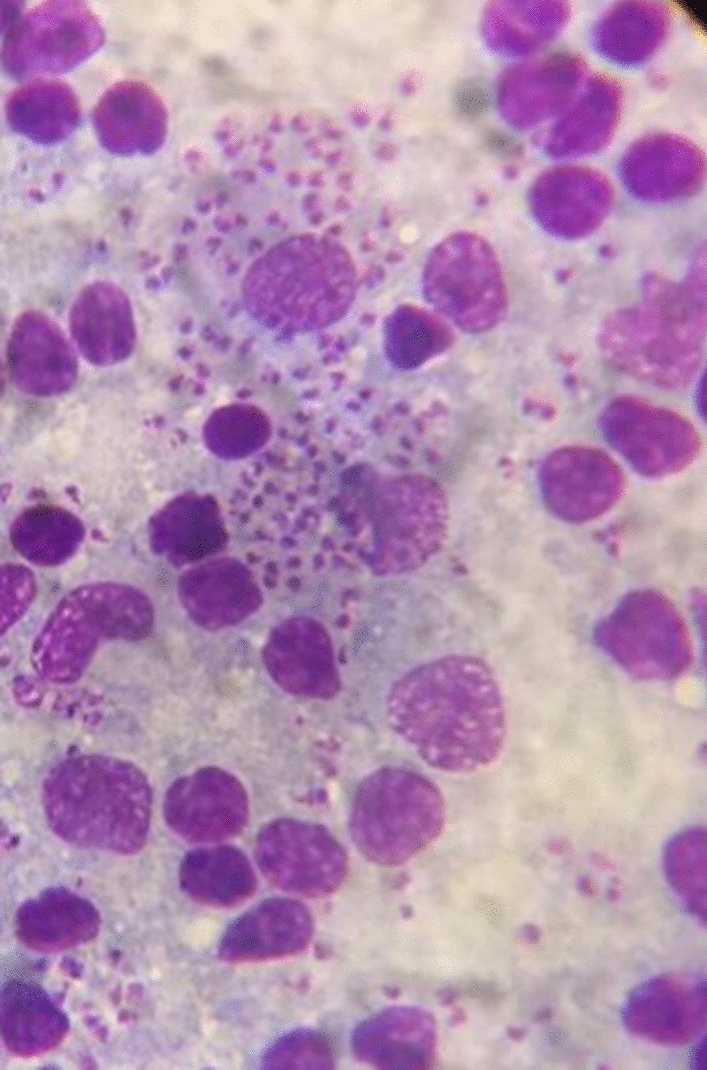

**Supplementary Information:**

The online version contains supplementary material available at 10.1186/s13071-021-04799-y.

## Background

Canine leishmaniosis (CanL) is an endemic disease in many countries throughout the world [1–3]. It is mainly caused by *Leishmania infantum* [4] and infected dogs may either control the infection and remain clinically healthy or develop variable clinical signs and/or be found to have abnormal laboratory test results [5].

The diagnosis of CanL is based on exposure history, clinical signs, immunodiagnostic techniques and/or parasite isolation [3, 6, 7]. The diagnostic tools for identifying *L. infantum* infection include direct techniques, such as parasitological (e.g. cytological and histological examination) and molecular (e.g. real-time PCR) assays, and indirect techniques, such as serum antibody detection methods (e.g. immunofluorescence antibody test [IFAT], enzyme-linked immunosorbent assay [ELISA] and/or immunochromatography tests [ICT]) [3, 6, 7].

Based on the clinical signs of the disease, laboratory test abnormalities and serology titres, the LeishVet group suggested a staging system which classifies the disease into four stages of evolution [6, 8]: mild (stage I), moderate (stage II, including substages A and B), severe (stage III) and very severe (stage IV) disease.

In terms of treatment, infected but clinically healthy dogs do not need immediate treatment against the parasite [9], but they should be monitored to assess potential seroconversion and the progression of infection towards disease [6, 10]. In contrast, sick animals should be treated in line with recommended antileishmanial protocols [6, 11]. The most recommended treatments consist of a combination of allopurinol and meglumine antimoniate (MA), or of allopurinol and miltefosine [3, 8, 11]. The administration of domperidone as part of the preventive protocols applied to healthy dogs and as immunotherapeutic drug in mild disease has been recently recommended as well [8, 12, 13].

Preventive measures should be applied to any infected dog to reduce the spread of infection [10]. The use of insecticides with a repellent effect is highly recommended [10, 14]. Healthy seronegative dogs may also be vaccinated to stimulate their immune response and prevent disease development upon infection [8, 10, 15, 16]. Domperidone, an immune modulator, has been studied [12, 13] and recommended as part of the preventive approach [8, 10].

Current trends in the diagnosis and treatment of CanL among veterinarians have been widely assessed in Spain [17–25]. However, few studies have been conducted in Portugal [19, 23, 26], and it is not known whether Portuguese veterinary practitioners follow the international recommendations or if these protocols vary according to the different stages of disease severity.

This study aimed to assess Portuguese veterinarians’ medical approach to CanL in terms of diagnosis, antileishmanial treatment and prevention protocols, taking into account various different theoretical scenarios/stages of CanL. A secondary aim was to ascertain whether the current guidelines are known to and followed by those practitioners.

## Methods

### Survey development and distribution

A questionnaire was developed using an online platform (Google Forms®). Several topics were assessed, including: (i) general characteristics of the veterinarian (5 items); (ii) the preferred protocols used for diagnosis (19 items), treatment (11 items) and prevention (2 items) of CanL, considering different theoretical scenarios of infection and disease, as well as the preferred tools for a general daily routine diagnosis; and (ii) current knowledge and application of the existing guidelines for the treatment of CanL (8 items) (Additional file 1: Table S1). The theoretical scenarios were designed to represent dogs infected with *L. infantum*, showing different manifestations consistent with subclinical infection, as well as clinical leishmaniosis in stages II (IIA and IIB) III and IV of the LeishVet clinical classification system.

The scenario representing a subclinical infection consisted of the case of a healthy 5-year-old male dog living in a CanL endemic area that was brought for consultation to the veterinarian for vaccination and which was found to be positive for anti-*Leishmania* antibodies (in 1:80 dilution) in a screening test. Physical examination, complete blood count (CBC) and biochemical profile testing revealed no abnormalities.

The scenario of a stage IIa infection consisted of the case of an 8-year-old spayed female dog which, 6 months prior to the consultation, had travelled to a CanL endemic region without any protection being provided against *Leishmania* spp. infection/disease. During the consultation the dog presented with lethargy and weight loss, and physical examination revealed periorbital alopecia, footpad exfoliative dermatitis and generalised lymphadenomegaly. In addition, mild non-regenerative anaemia, as well as hyperproteinaemia with hypoalbuminemia, hyperglobulinemia and polyclonal gammopathy were detected; however, renal parameters, such as serum creatinine urinalysis and urinary protein/creatinine ratio (UPC), were normal.

The scenario for the stage IIb CanL case was represented by a 6-year-old male dog living in a CanL endemic area, which presented with epistaxis, mild non-regenerative anaemia, hyperglobulinemia without hypoalbuminemia, serum creatinine level of 1.4 mg/dL and UPC of 0.5 (inactive sediment). High positive serological titres of anti-*Leishmania* antibodies were detected (1:640 dilution). This dog also had normal creatinine values (< 1.4 mg/dL) and an UPC of 0.5.

The scenario for the stage III case consisted of a 7-year-old male dog living in a CanL endemic area which was brought to the veterinarian due to lethargy, anorexia, weight loss, polyuria, polydipsia and auricular lesions. Physical examination revealed pale mucosae, generalised lymphadenomegaly, mucocutaneous ulcerative lesions, ear crusts, as well as ocular lesions compatible with blepharitis and uveitis. Complete blood count (CBC) showed moderate nonregenerative anaemia, and the biochemical profile and serum protein electrophoresis revealed azotemia (creatinine 1.9 mg/dl), hyperproteinaemia, hypoalbuminemia and hyperglobulinemia with polyclonal gammopathy. Furthermore, urinalysis showed proteinuria (UPC 1.2) and inappropriately concentrated urine (urinary specific gravity 1018), and high serological anti-*Leishmania* antibody titres were detected (1:320 dilution). Finally, ultrasound detected signs of splenomegaly. The systolic blood pressure was normal.

Finally, stage IV was represented by a 12-year-old male dog living in a CanL endemic area which presented with several clinical signs, such as lethargy, anorexia, weight loss, skin wounds, polyuria, polydipsia, pale mucosae, facial and plantar exfoliative dermatitis, onychogryphosis, nasal hyperkeratosis and ulceration and corneal opacification. Laboratory testing revealed this patient to also have moderate non-regenerative anaemia, as well as hyperproteinaemia, hypoalbuminemia and hyperglobulinemia with polyclonal gammopathy. Additionally, severe azotaemia (creatinine 3.5) and proteinuria (UPC 6.2) were detected, and IFAT serology resulted in a positive result for anti-*Leishmania* antibody titres at 1:640 dilution.

A sixth clinical case focused on the preventive approach to apply to a healthy 3-year-old male dog moving from a non-endemic to an endemic zone whose owners wanted disease screening and treatment with an adequate prophylaxis against leishmaniosis.

The diagnostic approach was surveyed, bearing in mind not only the different theoretical clinical cases but also, through other questions, factors relating to general daily practice, regardless of any specific clinical manifestation.

All scenarios described dogs that were living in, moving to or coming from an endemic area which had not been given any preventive measure for CanL, such as vaccination, insecticides/repellents or immunomodulators. The descriptions of the secenarios/cases aimed at highly suggesting a possible infection by *L. infantum*, with or without clinical signs, and thus guide the clinician towards the management of CanL. The descriptions of all clinical cases included information on the different serological titres of anti-*Leishmania* antibodies.

The preferred diagnostic tools for each stage and the need to perform additional testing for etiological diagnosis were surveyed. As the aim was to assess CanL management, it was assumed that the canine patients described in these theoretical scenarios were negative for other vector-borne disease pathogens, such as *Anaplasma* spp.,* Babesia* spp.,* Bartonella* spp.,* Borrelia* spp.,* Dirofilaria* spp., *Erlichia* spp., *Haemobartonella* spp.,* Hepatozoon* spp.,* Rickettsia* spp. and *Trypanosoma* spp. The order of presentation of the clinical cases was randomised, with the stage III case presented first, followed by the subclinical infection case, the stages IIa, IIb and IV cases, respectively, and finally the clinical case describing a healthy dog moving to an endemic area.

For internal validation, the questionnaire was evaluated by an epidemiologist and primarily distributed exclusively through the mailing list of ‘Masked for Review’ for 4 weeks. In second phase, it was also diffused* via* Portuguese social network veterinary groups and kept online for another 4 weeks.

The questionnaire was anonymous and the respondents were informed about its use for research purposes.

### Data processing and statistical analysis

All data were collected using Google Forms® and downloaded in a database (Microsoft Excel 2016®; Microsoft Corp., Redmond, WA, USA) for descriptive statistical analysis. Since no major abnormalities were detected during the internal validation process, the questionnaire content used in both first and second phases was the same and answers from ‘Masked for Review’ were included in the global descriptive statistical analysis.

## Results

**Fig. 1:**
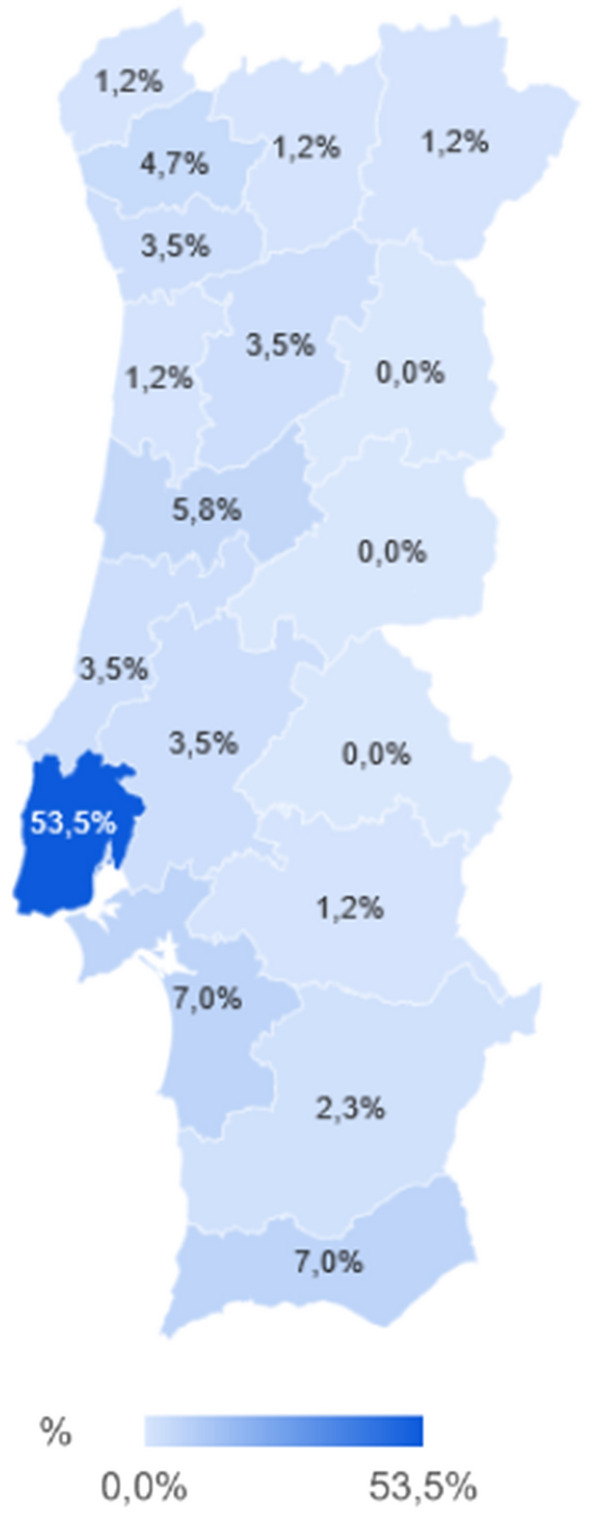
Geographical distribution of the surveyed clinicians

A total of 86 replies were obtained from clinicians working in 15 of the 20 geographical districts/island autonomous regions from northern to southern Portugal (Fig. 1). Replies were not obtained from veterinarians working in the continental districts of Castelo Branco, Guarda, Portalegre and the island autonomous regions of Azores and Madeira.

### Diagnostic approach considering different theoretical scenarios

**Fig. 2:**
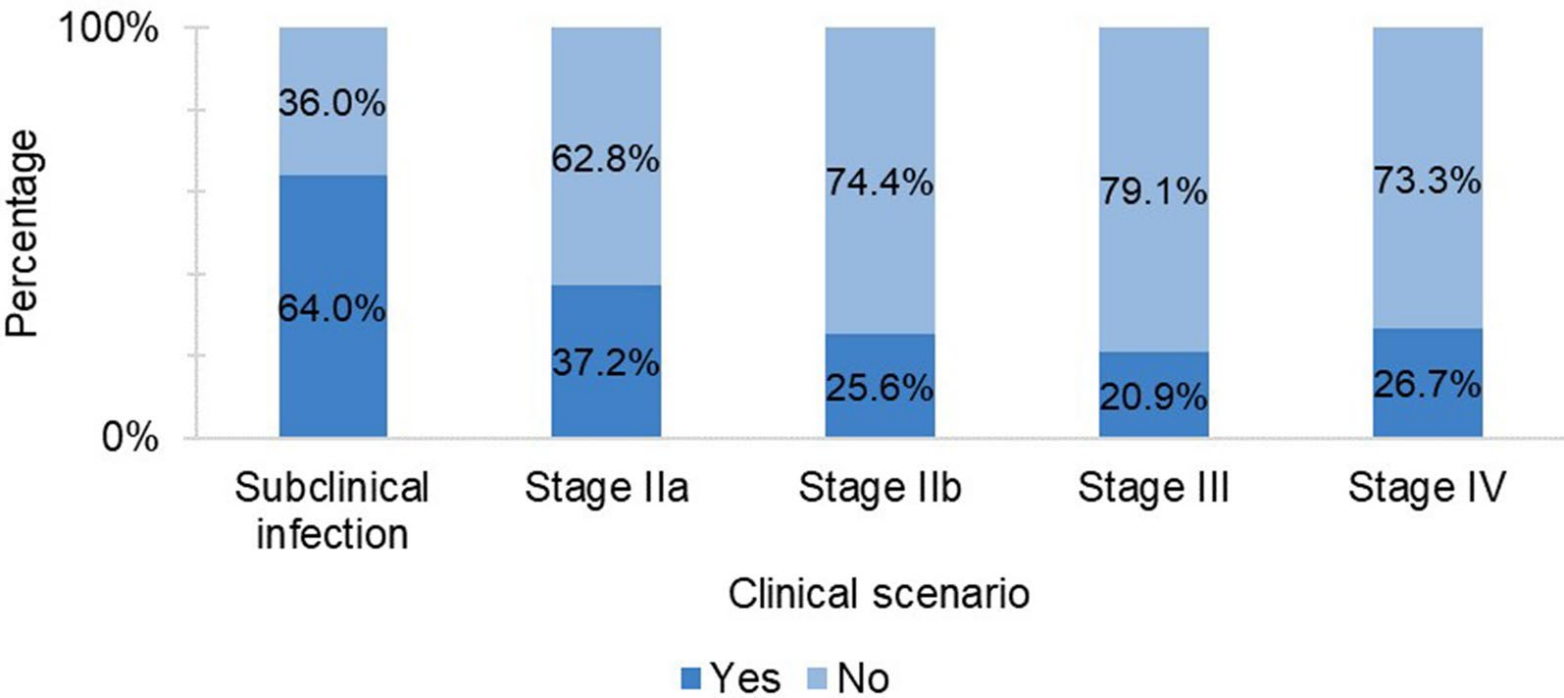
Percentage of responding veterinarians ( *n* = 86) deciding to perform/not perform additional complementary diagnostic tests according to the different scenarios of canine leishmaniosis

Taking into account the clinical signs, clinicopathological findings and serological titres mentioned for every clinical scenario, the willingness of veterinarians to use further diagnostic methods was surveyed. Of the 86 veterinary practitioners who responded, 64.0% (55/86) reported that they would require further diagnostic tests when faced with the scenario of subclinical infection; this proportion was 37.2% (32/86) for stage IIa, 25.6% (22/86) for stage IIb, 20.9% (18/86) for stage III and 26.7% (23/86) for stage IV (Fig. 2).

### First-choice diagnostic tools according to different theoretical scenarios

Among the respondents, 64.0% (55/86) reported that they would proceed with further tests to confirm infection in an infected but clinically healthy dog, of whom 47.3% (26/55) chose the PCR test on bone marrow (BM) or lymph nodes (LN), followed by the PCR test on blood (21.8%; 12/55), with about 14.5% (8/55) also mentioning serum protein electrophoresis. Cytology on BM, cytology on LN and other non-etiological tests were mentioned as well, in minor proportions (Table 1).

**Table 1:** Diagnostic tools used according to the different theoretical scenarios of suspected canine leishmaniosis

Diagnostic tools	Subclinical infection (*n* = 55)	Sstage IIa CanL (*n* = 32)	Stage IIb CanL (*n* = 22)	Stage III CanL (*n* = 18)	Stage IV CanL (*n* = 23)
Technique	*n* (%)				
Lymph node (LN) cytology	2 (3.6%)	14 (43.8%)	1 (4.5%)	11 (61.1%)	3 (13.0%)
Bone marrow (BM) cytology	3 (5.5%)	2 (6.3%)	2 (9.1%)	2 (11.1%)	2 (8.7%)
PCR test on BM/LN	26 (47.3%)	9 (28.1%)	5 (22.7%)	2 (11.1%)	2 (8.7%)
PCR test on blood	12 (21.8%)	2 (6.3%)	2 (9.1%)	0 (0.0%)	1 (4.3%)
“Other” diagnostic tests (one or more options):	12 (21.8%)^a^	5 (15.6%)^a^	12 (54.5%)^a^	3 (16.7%)^a^	15 (65.2%)^a^
Serum protein electrophoresis	8 (14.5%)		2 (9.1%)^b^	1 (5.6%)^b^	
Repeat serology testing in 2–3 months	3 (5.5%)				
LN cytology (combined with other examinations)	1 (1.8%)				1 (4.3%)
PCR testing on both blood and BM/LN	1 (1.8%)				
Exclusion of other skin parasitic infections		2 (6.3%)			
Exclusion of dirofilariosis			1 (4.6%)^a^		
Measurement of systolic blood pressure		1 (3.1%)	1 (4.6%)		2 (8.7%)
Exploring the nasal cavity (rhinoscopy)			2 (9.1%)		
Coagulation tests			2 (9.1%)		
Thoracic radiography			1 (4.6%)	1 (5.6%)	2 (8.7%)
Abdominal ultrasound		1 (3.1%)	3 (13.6%)	1 (5.6%)^2^	14(60.9%)
Urinalysis				1 (5.6%)^2^	
Urine culture				1 (5.6%)	2 (8.7%)
Complete blood count and/or biochemical parameters		2 (6.3%)^b^	1 (4.6%)^b^		1 (4.3%)^b^

*CanL* Canine leishmaniosis

*LN* lymph node(s)

*BM* bone marrow

*PCR* polymerase chain reaction

*SBP* systolic blood pressure

^a^Some respondents choosing “Other” diagnostic tests detailed more than one diagnostic tool. Therefore, the sum of those alternative techniques is often higher than the total

^b^Tests which were detailed in the description of that clinical case in the questionnaire

For the stage IIa CanL scenario, 37.2% (32/86) of responding veterinarians assumed that they would perform more diagnostic tests to confirm diagnosis in addition to serology testing, of whom 43.8% (14/32) chose LN cytology and 28.1% (9/32) preferred PCR testing on BM or LN. Among the remaining tests reported, cytological examination of BM and PCR testing of blood and other non-etiological tests were mentioned by a few respondents. For the CanL stage IIb scenario, 22.7% (5/22) or respondents reported they would perform PCR testing on blood to confirm diagnosis, and over one half (54.5%; 12/22) reported electing other non-etiologic complementary exams, with each technique being mentioned by few respondents (up to 13.6%, 3/22) (Table 1).

For the stage III CanL scenario, in addition to performing the clinical, laboratory and serology testing, 20.9% (18/86) of the respondents reported that they would carry out more tests to confirm CanL, of whom 61.1% (11/18) elected for LN cytology. Cytology examination of BM, PCR testing of BM/LN and other tests were chosen by 5.6% (1/18) to 11.1% (2/18) of respondents each (Table 1).

For the CanL case described in the stage IV CanL scenario, 26.7% (23/86) of responding veterinarians reported they would carry out additional examinations to confirm CanL, among whom 60.9% (14/23) would ask for an abdominal ultrasonography. Other diagnostic tests were evoked by 4.3% (1/23) to 13.0% (3/23) of respondents each (Table 1).

### Diagnostic tools and PCR matrix used on a routine basis

**Fig. 3:**
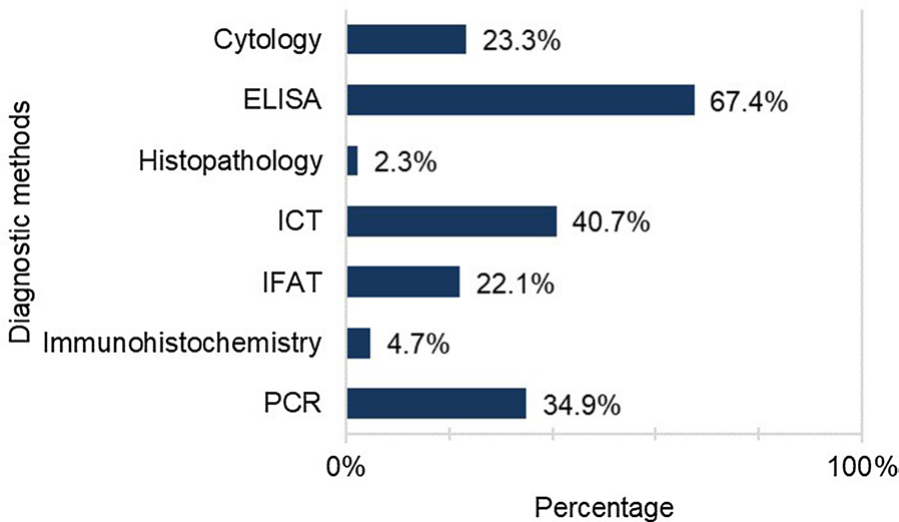
Diagnostic methods routinely used for detection of *Leishmania infantum* infection.* ELISA* Enzyme-linked immunosorbent assay,* ICT* immunochromatography,* IFAT* immunofluorescence antibody test. *n* = 86

Bearing in mind the preferred diagnostic techniques used on a general daily practice (i.e. regardless of any specific clinical scenario), ELISA was selected by 67.4% (58/86) of the respondents, followed by ICT tests (40.7%; 35/86), PCR tests (39.4%; 30/86), cytology (23.3%; 20/86), IFAT (22.1%; 19/86) and, in minor proportions, immunohistochemistry (4.7%; 4/86) and histopathology (2.3%; 2/86) (Fig. 3).

**Fig. 4:**
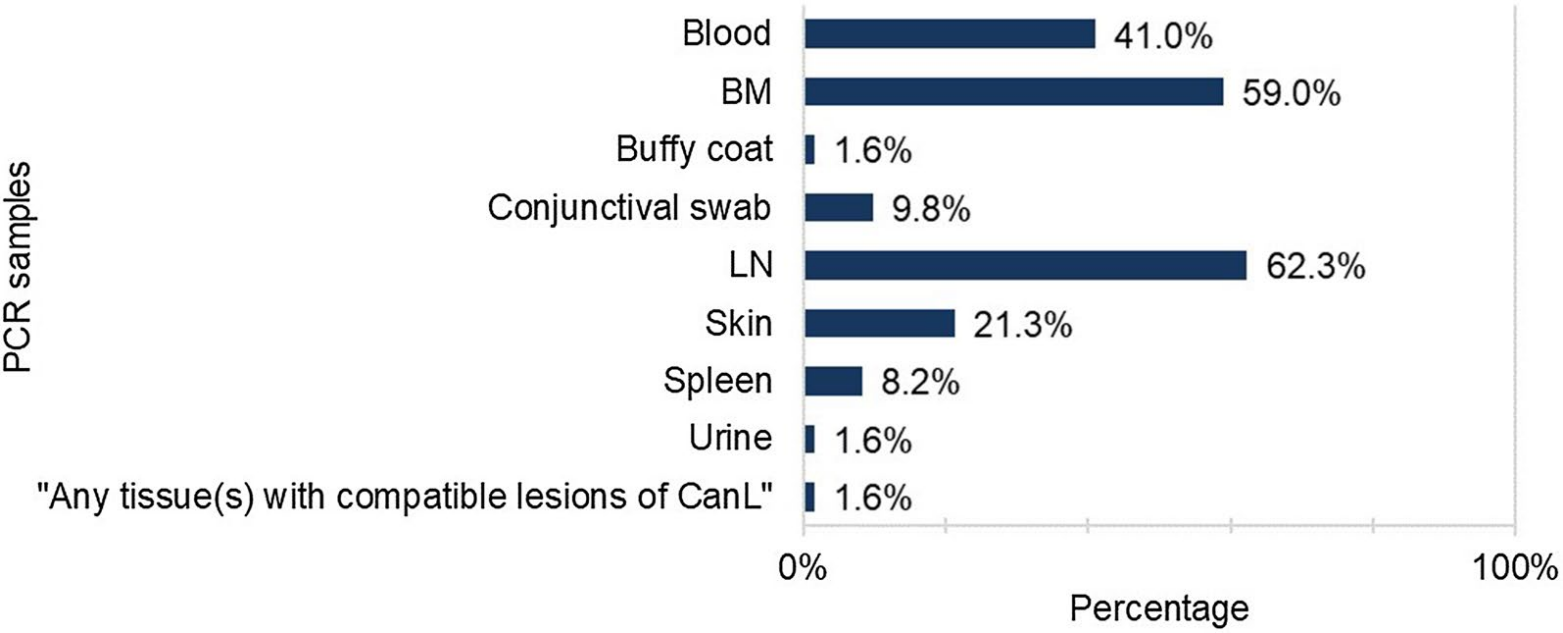
Choice of samples for PCR testing used by the 61 veterinarians who reported routinely using PCR testing for CanL.* BM* Bone marrow,* LN* lymph node(s),* CanL* canine leishmaniosis

Regarding the preferred matrix for the PCR test, 29.1% (25/86) of the responding veterinarians reported not using the PCR test on a routine basis. Of the 61 veterinarians who did perform PCR testing, the preference for LN matrix was evoked by 62.3% (38/61), followed by BM samples (59.0%; 36/61), blood (41.0%; 25/61) and skin (21.3%; 13/61) (Fig. 4). Conjunctival swabs (9.8%; 6/61), spleen aspirates (8.2%; 5/61), buffy coat (1.6%; 1/61), urine (1.6%; 1/61), or “any tissues containing lesions compatible with CanL” (1.6%; 1/61) were also selected as the test matrix by smaller percentages of respondents (Fig. 4).

### Antileishmanial treatment and euthanasia considering the different theoretical scenarios

For the subclinically infected dog, 51.2% (44/86) of the respondents would not apply any treatment; of the remaining respondents (48.8%; 42/86), 47.6% (20/42) elected for domperidone as a first-choice protocol, followed by monotherapy with allopurinol (23.8%; 10/42). Moreover, 28.6% (12/42) assumed performing associations of allopurinol with domperidone (19.0%; 8/42), miltefosine (7.1%; 3/42) or MA (2.4%; 1/42) (Table 2).

**Table 2:** First-choice treatment protocols for a dog suspected of subclinical CanL infection

Treatment protocol	*n* (%)
Allopurinol	10/42 (23.8)
Allopurinol + MA	1/42 (2.4)
Allopurinol + miltefosine	3/42 (7.1)
Allopurinol + domperidone	8/42 (19.0)
Domperidone	20/42 (47.6)

*MA* Meglumine antimoniate

**Fig. 5:**
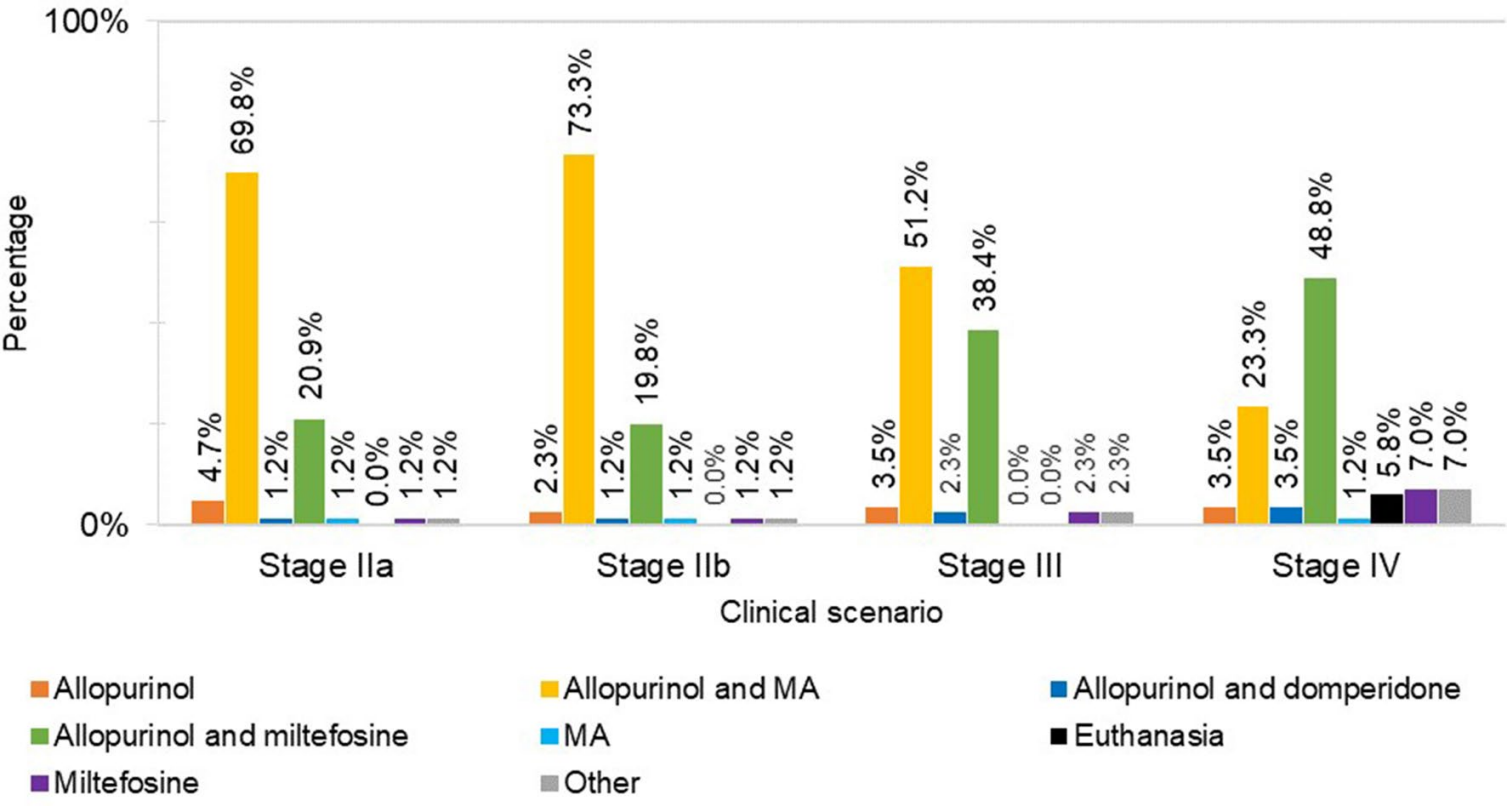
First-choice treatment protocols (or euthanasia) for dogs suspected having different stages of clinical CanL.* MA* Meglumine antimoniate

For dogs with stage IIa CanL, 69.8% (60/86) of respondents preferred allopurinol/MA and 20.9% (18/86) prioritised allopurinol/miltefosine. For dogs with stage IIb CanL, 73.3% (63/86) prescribed allopurinol/MA and 19.8% (17/86) used allopurinol/miltefosine. Protocols such as single therapy with allopurinol, MA or miltefosine, among others, were chosen by 9.3% (8/86) and 7.0% (6/86) of respondents for the treatment of stage IIa and IIb disease, respectively (Fig. 5).

In face of a scenario compatible with stage III CanL, 51.2% (44/86) assumed prescribing allopurinol/MA while 38.4% (33/86) preferred allopurinol/miltefosine. The remaining 10.5% (9/86) opted for other non-conventional protocols (Fig. 5).

For the theoretical scenario of a stage IV CanL infection, 72.1% of the respondents (62/86) preferred the combinations of allopurinol with MA, or allopurinol with miltefosine. More specifically, the allopurinol/miltefosine combination was more often selected (48.8%; 42/86) than the allopurinol/MA combination (23.3%; 20/86). Other protocols were evoked by 22.1% of respondents (19/86) (Fig. 5). This case scenario also had 5.8% (5/86) of the respondents opting for euthanasia, rather that applying any treatment.

### Prevention and control

From the 86 responding veterinarians, 98.8% admitted applying appropriate preventive measures and monitoring (including repeating serology testing within 3–6 months). The remaining 1.2% reported only monitoring patients.

For the case of the healthy dog moving to a CanL endemic area, the single use of insecticides/repellents was the choice of 5.8% (5/86), with the combination of insecticides/repellents with domperidone, vaccination or both reported by 16.3% (14/86), 54.7% (47/86) and 22.1% (19/86), respectively. The administration of vaccination as an exclusive treatment was chosen by 1.2% (1/86) (Table 3).

**Table 3:** Preventive protocols applied to a healthy dog moving to an endemic area

Preventive protocol	*n* (%)
Insecticides/repellents	5/86 (5.8)
Insecticides/repellents + domperidone	14/86 (16.3)
Insecticides/repellents + vaccination	47/86 (54.7)
Insecticides/repellents + domperidone + vaccination	19/86 (22.1)
Vaccination	1/86 (1.2)

### Awareness of guidelines on CanL

Among the 86 respondents, 93.0% (80) alleged to be aware of the existence of guidelines for the management of CanL, among whom 48.8% (39/80) alleged that they followed the LeishVet guidelines in their clinical practice and 15.0% (12/80) consulted those from the Canine Leishmaniosis Working Group (CLWG). The European Scientific Counsel Companion Animal Parasites (ESCCAP) and World Health Organisation (WHO) recommendations were evoked by 3.8% (3/80) and 1.3% (1/80) of the respondents, respectively. Although the remaining respondents (1.3%, (25/80) reported being aware of the existence of these guidelines, they stated that they did not apply any specific recommendations (Table 4).

**Table 4:** Guidelines accessed by the veterinarians for the management of CanL

Guidelines	*n* (%)
CLWG	12/80 (15.0%)
ESCCAP	3/80 (3.8%)
LeishVet	39/80 (48.8%)
WHO	1/80 (1.3%)
No guidelines applied	25/80 (31.3%)

*CLWG* Canine Leishmaniosis Working Group,* ESCCAP* European Scientific Counsel Companion Animal Parasites,* WHO* World Health Organisation

Those who followed the LeishVet guidelines were asked to stage each of the clinical cases according with those recommendations. The scenario representing a subclinical infection was classified as LeishVet stage I disease by 92.3% (36/39); those corresponding to stages IIa and IIb were considered as stage II by 84.6% (33/39) and 56.4% (22/39), respectively; and the scenarios representing stages III and IV were classified accordingly by 79.5% (31/39) and 92.3% (36/39), respectively.

## Discussion

The aim of this study was to scrutinise the main trends regarding the clinical approach to CanL, including diagnostic, antileishmanial treatment and prevention methods among general veterinary practitioners in Portugal, considering different scenarios of CanL. Overall, the results of our study showed that the need to perform further diagnostic tests generally decreased with the increasing severity of the clinicopathological findings.

The most widely used test to diagnose CanL in the general daily practice routine was the ELISA, followed by ICT rapid tests. These results are in contrast with those from previous surveys conducted in Portugal and other European countries, which found that IFAT or ICT is generally preferred to ELISA [19, 25, 26]. Even though IFAT has usually been accepted as the gold standard test [7, 27], ELISA has been found to have higher diagnostic performance than the other two tests [28], perhaps explaining its increasing use. Furthermore, IFAT requires a more subjective, operator-dependent interpretation than ELISA, and ICT tests provide only single qualitative data and reduced sensitivities, which have discouraged their use [6, 7, 29].

The use of PCR testing and cytological examination has apparently increased, compared with a previous survey conducted in Portugal that reported approximately 5–6% of veterinarians routinely using PCR and 2–10% using cytological tests [26]. Preference for immunohistochemistry and histopathology in our study was rare, similar to that reported in the earlier study [26]. Nevertheless, these techniques can help improve the diagnosis of CanL through providing detailed data and are widely used in research settings [7]. Our results may also reflect some lack of familiarity with such techniques. To our knowledge, this study is the first to report surveying the most used biological samples for *Leishmania* spp. DNA detection using PCR in Portuguese clinical practice. The preferred biological samples were LN and BM, which are widely considered to be among the most sensitive samples for testing [7, 8]. However, the frequent selection of blood samples may be explained by the fact that testing blood is an easier, less invasive and frequent procedure and that clinicians can use such samples to conduct further tests. The use of blood samples can also reflect some lack of awareness regarding scientific research updates and international recommendations that report blood as a less sensitive sample [7, 8].

With respect to the theoretical scenarios of CanL cases, over one half of the respondents would not treat a dog with subclinical infection, which is in line with the general recommendations which state that the monitoring of such dogs should be sufficient and that treatment could disrupt their immunological balance against infection [8, 9]. Among those who prescribed a medical treatment in this case, the majority used domperidone. There are several studies that have documented the use of domperidone not only as a preventive measure but also for the treatment of mild disease [8, 10, 30]. Therefore, the two preferred strategies (i.e. not treating or treating with domperidone) are in line with the LeishVet group guidelines [8]. The combined therapy of allopurinol/MA was the preferred protocol for the scenarios of stage II and III CanL, followed by the combination of allopurinol/miltefosine. Although both treatments have shown good effectiveness and safety [31–33], the allopurinol/MA association presented better results in a long-term study, associated with a faster recovery of clinical signs and improved laboratory findings, as well as fewer clinical relapses [31]. Nevertheless, both treatments are in line with the literature recommendations [8, 34] and international tendencies reported by some surveys [21, 23, 25]. The treatment protocols reported for the clinical scenario of stage IV CanL disease displayed a different trend from those for the remaining CanL stages, with the combined treatment of allopurinol/miltefosine being prioritised to that of allopurinol/MA. One explanation of these results may be that the use of MA has been associated to nephrotoxicity, as opposed to the use of miltefosine that has not [35]. However, it is debatable whether it is the treatment or the immune complex deposition that causes renal damage [31, 32]. Taking into account the severity of stage IV CanL disease, an individual approach is recommended [8, 34, 36]. In the present study, this stage was the only one for which euthanasia was considered as an option. Given that the main differences between the stage III and IV CanL clinical scenarios were the renal values and serological titres, these results may emphasise the importance of renal disease in the establishment of a prognosis and the decision to euthanise [37, 38].

When considering the stages II to IV CanL scenarios, a small percentage of respondents mentioned other treatments which are not recommended due to the lack of compelling scientific evidence of their efficacy and/or safety [34]. Some have even shown discouraging results, such as the potential nephrotoxicity of a single therapy with MA [35], the lower efficacy of monotherapies compared with combined protocols [31] or the development of drug resistance to allopurinol [39]. Although these non-scientific-supported protocols were only reported a minor percentage of respondents, the fact that they were mentioned reflects a lack of awareness and information of current treatment recommendations.

In terms of prevention and control strategies, nearly all respondents acted according to the international recommendations, employing both monitoring and preventive measures when presented with an infected but clinically healthy patient [6]. The use of insecticides/repellents in association with vaccination was the preferred protocol, followed by the combination of insecticides/repellents, vaccination and domperidone and, in third place, combined treatment with insecticides/repellents and domperidone. The administration of insecticide/repellent formulations, especially as spot-ons or impregnated collars, has been widely recognised as the most efficient and recommended method to prevent infection by *Leishmania* spp. [6, 10, 40]. However, to maximise prevention, a multimodal approach involving vaccination in association with insecticides/repellents is recommended in seronegative dogs [10]. Nevertheless, vaccination raises some controversy as certain vaccines may interfere with serological screening [15, 29], and no data are as yet available on the serological status such dogs. Domperidone was the least preferred option, but it was still the choice of over one third of respondents. This proportion is lower than that reported in Spain (45–50%) [21, 24], but higher than that found in France where only 1.6% of the practitioners prescribed domperidone [24]. Information on domperidone use in Portugal is scarce. To our knowledge, the only study conducted to date on domperidone usage in Portugal reported that less than 15% of Portuguese clinicians prescribe domperidone as a prophylactic measure [23]. Thus, the results from our study show an apparent increase in its prescribing. Domperidone has been the most recent compound showing promising results in stimulating the immune response and preventing the development of disease in infected dogs [12, 13, 41], but compelling research on its efficacy and safety is still lacking [42].

Regarding the awareness of responding veterinarians on international guidelines, our results show that by far the majority is aware of their existence, especially those guidelines from the LeishVet group [6, 8] and the CLWG [7, 11, 40]. However, the fact that almost one third of respondents admitted knowing of their existence but denied applying any guidelines should raise some concern, since these practitioners may be missing the most recent developments reported in these guidelines.

The present study has a number of limitations. The number of replies (*n* = 86) was relatively small relative to the number of veterinarians in the entire country. However, neither the proportion of small-animal clinicians consulting the network group in which the questionnaire was launched nor how many of these are actively working is none. Nevertheless, this number is in line with the response to similar surveys previously conducted in Portugal [23, 26]. Shorter, simpler questionnaires spread not only through social media but directly* via* email to various clinics might result in a higher coverage and allow calculation of the percentage of replies. Nevertheless, the complexity and the length of the questionnaire might have prevented some people to whom it was not directed, such as non-veterinarians or veterinarians not working in clinical practice, from responding.

In an international perspective, a larger study would be helpful not only to determine whether the current practical approach is similar among countries where CanL is endemic, but also for future comparative research. Only by studying the current practical trends of general veterinary practitioners can the scientific community establish the correct bridge between clinical research and daily practice, identifying potential flaws and sensitising the small-animal veterinary community for a better evidence-based medicine approach.

## Conclusions

In conclusion, the present study highlights the current trends in and practical approaches to theoretical scenarios of CanL in Portugal. Overall, most general practitioners who answered this survey follow the international guidelines recommendations and the state of the art for the clinical management of CanL.

## Supplementary Information

**Additional file 1**: **Table S1.** Questionnaire provided online to veterinarians: “Management of canine leishmaniosis in Portugal: questionnaire-based survey”.

## Data Availability

The dataset(s) supporting the conclusions of this article is(are) included within the article (and its additional file).

## Abbreviations

BM: Bone marrow
CanL: Canine leishmaniosis
CBC: Complete blood count
CLWG: Canine Leishmaniosis Working Group
ELISA: Enzyme-linked immunosorbent assay
ESCCAP: European Scientific Counsel Companion Animal Parasites
ICT: Immunochromatography
IFAT: Immunofluorescence antibody test
LN: Lymph node(s)
UPC: Urinary protein/creatinine ratio

## References

[1] Dantas-Torres F, Solano-Gallego L, Baneth G, Ribeiro VM, de Paiva-Cavalcanti M, Otranto D (2012). Canine leishmaniosis in the Old and New Worlds: unveiled similarities and differences. Trends Parasitol.

[2] Maia C, Cardoso L (2015). Spread of *Leishmania infantum* in Europe with dog travelling. Vet Parasitol.

[3] European Scientific Counsel Companion Animal Parasites (ESCCAP). Control of vector-borne diseases in dogs and cats. 2019. p. 9–17. https://www.esccap.org/guidelines/gl5/. Accessed 30 Dec 2020.

[4] Ross MR (1903). Further notes on leishman’s bodies. BMJ.

[5] Solano-Gallego L, Koutinas A, Miró G, Cardoso L, Pennisi MG, Ferrer L (2009). Directions for the diagnosis, clinical staging, treatment and prevention of canine leishmaniosis. Vet Parasitol.

[6] Solano-Gallego L, Miró G, Koutinas A, Cardoso L, Pennisi MG, Ferrer L (2011). LeishVet guidelines for the practical management of canine leishmaniosis. Parasites Vectors.

[7] Paltrinieri S, Gradoni L, Roura X, Zatelli A, Zini E (2016). Laboratory tests for diagnosing and monitoring canine leishmaniasis. Vet Clin Pathol.

[8] LeishVet. LeishVet guidelines for the practical management of canine and feline leishmaniosis: a brief for the practicing veterinarian. 2018. http://www.leishvet.org/wp-content/uploads/2018/09/EN-Guidelines.pdf. Accessed 2 Mar 2020.

[9] Miró G, López-Vélez R (2018). Clinical management of canine leishmaniosis versus human leishmaniasis due to *Leishmania infantum*: Putting “One Health” principles into practice. Vet Parasitol.

[10] Miró G, Petersen C, Cardoso L, Bourdeau P, Baneth G, Solano-Gallego L (2017). Novel areas for prevention and control of Canine Leishmaniosis. Trends Parasitol.

[11] Oliva G, Roura X, Crotti A, Maroli M, Castagnaro M, Gradoni L (2010). Guidelines for treatment of leishmaniasis in dogs. J Am Vet Med Assoc.

[12] Gómez-Ochoa P, Castillo JA, Gascón M, Zarate JJ, Alvarez F, Couto CG (2009). Use of domperidone in the treatment of canine visceral leishmaniasis: a clinical trial. Vet J.

[13] Sabaté D, Llinás J, Homedes J, Sust M, Ferrer L (2014). A single-centre, open-label, controlled, randomized clinical trial to assess the preventive efficacy of a domperidone-based treatment programme against clinical canine leishmaniasis in a high prevalence area. Prev Vet Med.

[14] Fondati A, Gradoni L, Lubas G, Paltrinieri S, Roura X, Zatelli A (2018). Leishmaniasis prevention: what should be known before recommending a topical product against sand fly bites in dogs?. Veterinaria.

[15] Moreno J, Vouldoukis I, Schreiber P, Martin V, Mcgahie D, Gueguen S (2014). Primary vaccination with the LiESP/QA-21 vaccine (CaniLeish®) produces a cell-mediated immune response which is still present 1 year later. Vet Immunol Immunopathol.

[16] Fernández Cotrina J, Iniesta V, Monroy I, Baz V, Hugnet C, Marañon F (2018). A large-scale field randomized trial demonstrates safety and efficacy of the vaccine LetiFend® against canine leishmaniosis. Vaccine.

[17] Ruiz de Ybáñez R, del Río L, Martínez-Carrasco C, Segovia M, Cox J, Davies C (2009). Questionnaire survey on canine leishmaniosis in southeastern Spain. Vet Parasitol..

[18] Gálvez R, Miró G, Descalzo MA, Molina R (2011). Questionnaire-based survey on the clinical management of canine leishmaniosis in the Madrid region (central Spain). Prev Vet Med.

[19] Bourdeau P, Saridomichelakis MN, Oliveira A, Oliva G, Kotnik T, Gálvez R (2014). Management of canine leishmaniosis in endemic SW European regions: a questionnaire-based multinational survey. Parasites Vectors.

[20] Ballart C, Alcover MM, Picado A, Nieto J, Castillejo S, Portús M (2013). First survey on canine leishmaniasis in a non classical area of the disease in Spain (Lleida, Catalonia) based on a veterinary questionnaire and a cross-sectional study. Prev Vet Med.

[21] Lladró S, Picado A, Ballart C, Portús M, Gállego M (2017). Management, prevention and treatment of canine leishmaniosis in north-eastern Spain: an online questionnaire-based survey in the province of Girona with special emphasis on new preventive methods (CaniLeish vaccine and domperidone). Vet Rec.

[22] Alcover MM, Ballart C, Serra T, Castells X, Scalone A, Castillejo S (2013). Temporal trends in canine leishmaniosis in the Balearic Islands (Spain): a veterinary questionnaire. Prospective canine leishmaniosis survey and entomological studies conducted on the Island of Minorca, 20 years after first data were obtained. Acta Trop..

[23] Mattin MJ, Solano-Gallego L, Dhollander S, Afonso A, Brodbelt DC (2014). The frequency and distribution of canine leishmaniosis diagnosed by veterinary practitioners in Europe. Vet J.

[24] le Rutte EA, van Straten R, Overgaauw PAM (2018). Awareness and control of canine leishmaniosis: a survey among Spanish and French veterinarians. Vet Parasitol.

[25] Montoya A, Gálvez R, Checa R, Sarquis J, Plaza A, Barrera JP (2020). Latest trends in *L. infantum* infection in dogs in Spain, Part II: Current clinical management and control according to a national survey of veterinary practitioners. Parasites Vectors..

[26] Oliveira AM, Diaz S, Santos C, Bourdeau P, Pereira I (2010). Geographical distribution, clinical presentation, treatment and prevention of canine leishmaniosis in Portugal: a 2007 field survey. Rev Port Ciênc Vet.

[27] World Organisation for Animal Health (OIE). https://www.oie.int/fileadmin/Home/eng/Health_standards/tahm/3.01.11_LEISHMANIOSIS.pdf. Accessed 29 Apr 2020.

[28] Solano-Gallego L, Villanueva-Saz S, Carbonell M, Trotta M, Furlanello T, Natale A (2014). Serological diagnosis of canine leishmaniosis: Comparison of three commercial ELISA tests (Leiscan®, ID Screen® and Leishmania 96®), a rapid test (Speed Leish K®) and an in-house IFAT. Parasites Vectors.

[29] Solano-Gallego L, Cardoso L, Pennisi MG, Petersen C, Bourdeau P, Oliva G (2017). Diagnostic challenges in the era of canine *Leishmania infantum* vaccines. Trends Parasitol.

[30] Sabaté D, Llinás J, Homedes J, Sust M, Ferrer L (2014). A single-centre, open-label, controlled, randomized clinical trial to assess the preventive efficacy of a domperidone-based treatment programme against clinical canine leishmaniasis in a high prevalence area. Prev Vet Med..

[31] Manna L, Corso R, Galiero G, Cerrone A, Muzj P, Gravino AE (2015). Long-term follow-up of dogs with leishmaniosis treated with meglumine antimoniate plus allopurinol versus miltefosine plus allopurinol. Parasites Vectors.

[32] Santos MF, Alexandre-Pires G, Pereira MA, Marques CS, Gomes J, Correia J (2019). Meglumine antimoniate and miltefosine combined with allopurinol sustain pro-inflammatory immune environments during canine leishmaniosis treatment. Front Vet Sci..

[33] Miró G, Oliva G, Cruz I, Cañavate C, Mortarino M, Vischer C (2009). Multicentric, controlled clinical study to evaluate effectiveness and safety of miltefosine and allopurinol for canine leishmaniosis. Vet Dermatology.

[34] Roura X, Cortadellas O, Day MJ, Benali SL, D’Anna N, Fondati A, et al. Canine leishmaniosis and kidney disease: Q&A for an overall management in clinical practice. J Small Anim Pract. 2020. https://doi.org/10.1111/jsap.13237. 10.1111/jsap.13249 33169394

[35] Bianciardi P, Brovida C, Valente M, Aresu L, Cavicchioli L, Vischer C (2009). Administration of miltefosine and meglumine antimoniate In healthy dogs: clinicopathological evaluation of the impact on the kidneys. Toxicol Pathol.

[36] International Renal Interest Society (IRIS). Treatment recommendations for CKD in dogs 2019. http://www.iris-kidney.com/pdf/IRIS-DOG-Treatment_Recommendations_2019.pdf. Accessed 29 Apr 2020.

[37] Geisweid K, Mueller R, Sauter-Louis C, Hartmann K (2012). Papers: Prognostic analytes in dogs with *Leishmania infantum* infection living in a non-endemic area. Vet Rec.

[38] Pereira MA, Santos R, Oliveira R, Costa L, Prata A, Gonçalves V (2020). Prognostic factors and life expectancy in canine leishmaniosis. Vet Sci.

[39] Yasur-Landau D, Jaffe CL, David L, Baneth G (2016). Allopurinol Resistance in *Leishmania infantum* from dogs with disease relapse. PLoS Negl Trop Dis..

[40] Maroli M, Gradoni L, Oliva G, Castagnaro M, Crotti A, Lubas G (2010). Guidelines for prevention of leishmaniasis in dogs. J Am Vet Med A.

[41] Gómez-Ochoa P, Sabate D, Homedes J, Ferrer L (2012). Use of the nitroblue tetrazolium reduction test for the evaluation of Domperidone effects on the neutrophilic function of healthy dogs. Vet Immunol Immunopathol.

[42] Travi BL, Miró G (2018). Use of domperidone in canine visceral leishmaniasis: gaps in veterinary knowledge and epidemiological implications. Mem Inst Oswaldo Cruz..

