# Supplementary material for: Diagnosis and clinical management of canine leishmaniosis by general veterinary practitioners: a questionnaire-based survey in Portugal
Source: Parasit Vectors. 2021 Jun 7;14:306. doi: 10.1186/s13071-021-04799-y (PMC8182999; doi:10.1186/s13071-021-04799-y)
Supplement: Supplementary file 1 — Additional file 1: Table S1. Questionnaire provided online to veterinarians: “Management of canine leishmaniosis in Portugal: questionnaire-based survey”. [file 13071_2021_4799_MOESM1_ESM.docx]

**Questionnaire provided online – English version**

**Management of canine Leishmaniosis in Portugal – diagnosis and treatment**

Hello! If you are a veterinarian working in Portugal, in small animal clinics – namely, with the canine species – then, this questionnaire is for you!

This questionnaire is part of a *masked for review* approaching the diagnosis and treatment of Canine Leishmaniosis in Portugal.

It consists, essentially, of three parts: “Veterinarian profile”, “Clinical cases” and “Other questions”, always related to canine leishmaniosis.

The questionnaire contains around 50 questions and will take approximately 15 minutes to answer. It is worth noting that the questions are anonymous and will be analysed with proper confidentially, thus we kindly ask you to answer honestly, to allow obtaining valid and representative conclusions. Data privacy will be equally preserved in case of those being used in written or oral communications, or in publications that may result from the study.

In case of any questions related to this questionnaire, please contact, via email, the student *masked for review* or *masked for review*.

More information concerning the study will be sent to the participants, via email, if it is their willing. In that case, please send an email showing your interest to *masked for review.*

Thank you for your collaboration and time spent replying this questionnaire!

**FIRST PART – VETERINARIAN PROFILE**

1. Which is your academic degree in veterinary medicine? Select the highest you have, among the following.

□ Doctor of Philosophy (PhD) in Veterinary Sciences

□ Doctor in Veterinary Medicine (DVM) (pre-Bolonha)

□ Master of Science (MSc) (post-Bolonha)

1. How many years of clinical practice with dogs do you have?

□ Less than 2 □ 2 to 5 □ 6 to 10

□ 11 to 15 □ 16 to 20 □ More than 20

1. Select the district in which you mostly work as a veterinarian.

(List of Portuguese districts).

1. The type of area you work in, is:

□ Rural □ Urban □ Both

1. Approximately, how many confirmed cases of leishmaniosis did you have in the past 12 months?

□ Less than 5 □ 5 to 10 □ 11 to 15 □ 16 to 20 □ More than 20

**SECOND PART – CLINICAL CASES**

Six clinical cases will be presented, always related to canine leishmaniosis. Answers should be given bearing in mind your clinical practice context and knowledge on this topic.

**1^st^ Clinical case**

Dog, male, 7 years old, living in a geographic area where leishmaniosis is endemic and does not follow any prevention measures for the disease (repellents, vaccine, immunomodulators).

**Reason for consultation**: prostration, anorexia, weight loss, polyuria/polydipsia, auricular lesions.

**General examination:** Pale mucosae, generalised lymphadenomegaly, mucocutaneous ulcerative lesions, ears’ crusts.

**Ophthalmologic exam:** blepharitis, uveitis (bilateral intraocular pressure reduction, erythema).

**Abdominal ultrasound:** splenomegaly.

**Laboratory tests:** Moderate nonregenerative anaemia, hyperproteinaemia, hypoalbuminemia, hyperglobulinemia with polyclonal gammopathy.

Mild azotaemia (creatinine: 1.9mg/dL); urinalysis: USG: 1.018, proteinuria (RPCU: 1.2) with inactive sediment.

Normal systemic blood pressure.

**Serology (immunofluorescence):**

Anti-*Leishmania* antibody titre: positive for 1:320 dilution.

Negative serology for other concomitant vectorial agents (*Anaplasma* spp*., Babesia* spp.*, Ehrlichia* spp.*, Borrelia* spp.*, Rickettsia* spp.*, Hepatozoon* spp.*, Hemobartonella canis* spp.*, Bartonella* spp.*, Dirofilaria* spp.*, Trypanosoma* spp.).

**Reference serological values:**

Cut-off: positive anti-𝘓𝘦𝘪𝘴𝘩𝘮𝘢𝘯𝘪𝘢 antibody titres at 1:80 dilution.

1. Admitting leishmaniosis as one of the main differential diagnostics in this case, would you consider necessary doing more complementary diagnostic exams?

□ No □ Yes

1. If yes, which one would you prefer to do?

□ Lymph node(s) cytology

□ Bone marrow cytology

□ *Leishmania* PCR on spleen

□ *Leishmania* PCR on bone marrow or lymph node(s)

□ *Leishmania* PCR on blood

□ Other

1. If you selected “Other”, which one is it?
2. Which treatment would you do in this case?

| □ Allopurinol  □ Allopurinol and nucleotide analogues  □ Allopurinol and meglumine antimoniate  □ Allopurinol and domperidone  □ Allopurinol and miltefosine  □ Nucleotide analogues and active hexose correlated compounds (AHCC) | □ Meglumine antimoniate  □ Domperidone  □ Eutanasia  □ Miltefosine  □ Other |
| --- | --- |

1. In case you selected “Other”, which treatment is it?

**2^nd^ Clinical case**

Dog, male, 5 years old, living in a geographic area where leishmaniosis is endemic and does not follow any prevention measures for the disease (repellents, vaccine, immunomodulators).

**Reason for consultation**: vaccination.

**General examination:** No abnormalities.

**Laboratory tests:** CBC, biochemical profile and urinalysis without abnormalities.

**Serology (immunofluorescence):**

Anti-*Leishmania* antibody titre: positive for 1:80 dilution.

Negative serology for other concomitant vectorial agents (*Anaplasma* spp*., Babesia* spp.*, Ehrlichia* spp.*, Borrelia* spp.*, Rickettsia* spp.*, Hepatozoon* spp.*, Hemobartonella canis* spp.*, Bartonella* spp.*, Dirofilaria* spp.*, Trypanosoma* spp.*)*.

**Reference serological values:**

Cut-off: positive anti-𝘓𝘦𝘪𝘴𝘩𝘮𝘢𝘯𝘪𝘢 antibody titres at 1:80 dilution.

1. Would you consider necessary to perform more complementary exams in this case?

□ No □ Yes

1. If yes, which one would you prefer to do?

□ Lymph node(s) cytology

□ Bone marrow cytology

□ *Leishmania* PCR on bone marrow or lymph node(s)

□ *Leishmania* PCR on blood

□ Other

1. In case you selected “Other”, which one is it?
2. Would you treat this case?

□ No □ Yes

1. If yes, which treatment would you do?

| □ Allopurinol  □ Allopurinol and nucleotide analogues  □ Allopurinol and meglumine antimoniate  □ Allopurinol and domperidone  □ Allopurinol and miltefosine  □ Nucleotide analogues and active hexose correlated compounds (AHCC) | □ Meglumine antimoniate  □ Domperidone  □ Miltefosine  □ Other |
| --- | --- |

1. In case you selected “Other”, which treatment is it?
2. Regardless of applying treatment or not, would you monitor and/or apply preventive measures in this case?

□ Only clinical monitoring (including repeating serology in 3-6 months)

□ Only prevention against leishmaniosis, adequate to the clinical case

□ Adequate prevention and clinical monitoring (including repeating serology in 3-6 months)

**3^rd^ Clinical case**

Dog, female, 8 years old, spayed. Travelled 6 months before to an area where leishmaniosis is endemic but did not perform any preventive measure for the disease (repellent, vaccination or immunomodulators) before or during the trip.

**Reason for consultation:** lethargy and weight loss.

**General examination:** Periorbital alopecia, footpad exfoliative dermatitis, generalised lymphadenomegaly.

**Laboratory tests:** Mild nonregenerative anaemia, hyperproteinaemia, hypoalbuminemia, hyperglobulinemia with polyclonal gammopathy. No azotaemia neither proteinuria.

**Serology (immunofluorescence):**

Anti-*Leishmania* antibody titre: positive for 1:160 dilution.

Negative serology for other concomitant vectorial agents (*Anaplasma* spp*., Babesia* spp.*, Ehrlichia* spp.*, Borrelia* spp.*, Rickettsia* spp.*, Hepatozoon* spp.*, Hemobartonella canis* spp.*, Bartonella* spp.*, Dirofilaria* spp.*, Trypanosoma* spp.*)*.

**Reference serological values:**

Cut-off: positive anti-𝘓𝘦𝘪𝘴𝘩𝘮𝘢𝘯𝘪𝘢 antibody titres at 1:80 dilution.

1. Admitting leishmaniosis as one of the main differential diagnostics in this case, would you consider necessary doing more complementary diagnostic exams?

□ No □ Yes

1. If yes, which one would you prefer to do?

□ Lymph node(s) cytology

□ Bone marrow cytology

□ *Leishmania* PCR on bone marrow or lymph node(s)

□ *Leishmania* PCR on blood

□ Other

1. In case you selected “Other”, which one is it?
2. Assuming a definitive diagnosis of leishmaniosis, which treatment would you apply?

| □ Allopurinol  □ Allopurinol and nucleotide analogues  □ Allopurinol and meglumine antimoniate  □ Allopurinol and domperidone  □ Allopurinol and miltefosine  □ Nucleotide analogues and active hexose correlated compounds (AHCC) | □ Meglumine antimoniate  □ Domperidone  □ Miltefosine  □ Other |
| --- | --- |

1. In case you selected “Other”, which treatment is it?

**4th clinical case**

Dog, male, 6 years old. Lives in a geographic area where leishmaniosis is endemic and does not follow any prevention measures for the disease (repellents, vaccine, immunomodulators).

**Reason for consultation**: Epistaxis.

**General examination:** No abnormalities.

**Laboratory tests:** Mild nonregenerative anaemia. Hyperglobulinaemia without hypoalbuminemia. Creatinine <1.4 mg/dL (normal), borderline proteinuria (RPCU: 0.5), inactive sediment.

**Serology (immunofluorescence):**

Anti-*Leishmania* antibody titre: positive for 1:640 dilution.

Negative serology for other concomitant vectorial agents (*Anaplasma* spp*., Babesia* spp.*, Ehrlichia* spp.*, Borrelia* spp.*, Rickettsia* spp.*, Hepatozoon* spp.*, Hemobartonella canis* spp.*, Bartonella* spp.*, Dirofilaria* spp.*, Trypanosoma* spp.*)*.

**Reference serological values:**

Cut-off: positive anti-𝘓𝘦𝘪𝘴𝘩𝘮𝘢𝘯𝘪𝘢 antibody titres at 1:80 dilution.

1. Admitting leishmaniosis as one of the main differential diagnostics in this case, would you consider necessary doing more complementary diagnostic exams?

□ No □ Yes

1. If yes, which one would you prefer to do?

□ Lymph node(s) cytology

□ Bone marrow cytology

□ *Leishmania* PCR on bone marrow or lymph node(s)

□ *Leishmania* PCR on blood

□ Other

1. In case you selected “Other”, which one is it?
2. Assuming a definitive diagnosis of leishmaniosis, which treatment would you apply?

| □ Allopurinol  □ Allopurinol and nucleotide analogues  □ Allopurinol and meglumine antimoniate  □ Allopurinol and domperidone  □ Allopurinol and miltefosine  □ Nucleotide analogues and active hexose correlated compounds (AHCC) | □ Meglumine antimoniate  □ Domperidone  □ Eutanasia  □ Miltefosine  □ Other |
| --- | --- |

1. In case you selected “Other”, which treatment is it?

**5^th^ Clinical case**

Dog, male, 12 years old. Lives in a geographic area where leishmaniosis is endemic and does not follow any prevention measures for the disease (repellents, vaccine, immunomodulators).

**Reason for consultation**: lethargy, anorexia, weight loss, skin wounds, polyuria/polydipsia.

**General examination:** Pale mucosae, facial and plantar exfoliative dermatitis, corneal opacity, onychogryphosis, nasal hyperkeratosis and ulceration.

**Laboratory tests:** Moderate nonregenerative anaemia; hyperglobulinemia with polyclonal gammopathy, hypoalbuminemia. Azotaemia (creatinine: 3.5mg/dL), proteinuria (UPC: 6.2), inactive sediment.

**Serology (immunofluorescence):**

Anti-*Leishmania* antibody titre: positive for 1:640 dilution.

Negative serology for other concomitant vectorial agents (*Anaplasma* spp*., Babesia* spp.*, Ehrlichia* spp.*, Borrelia* spp.*, Rickettsia* spp.*, Hepatozoon* spp.*, Hemobartonella canis* spp.*, Bartonella* spp.*, Dirofilaria* spp.*, Trypanosoma* spp.*)*.

**Reference serological values:**

Cut-off: positive anti-𝘓𝘦𝘪𝘴𝘩𝘮𝘢𝘯𝘪𝘢 antibody titres at 1:80 dilution.

1. Admitting leishmaniosis as one of the main differential diagnostics in this case, would you consider necessary doing more complementary diagnostic exams?

□ No □ Yes

1. If yes, which one would you prefer to do?

□ Lymph node(s) cytology

□ Bone marrow cytology

□ *Leishmania* PCR on bone marrow or lymph node(s)

□ *Leishmania* PCR on blood

□ Other

1. In case you selected “Other”, which one is it?
2. Which treatment would you apply?

| □ Allopurinol  □ Allopurinol and nucleotide analogues  □ Allopurinol and meglumine antimoniate  □ Allopurinol and domperidone  □ Allopurinol and miltefosine  □ Nucleotide analogues and active hexose correlated compounds (AHCC) | □ Meglumine antimoniate  □ Domperidone  □ Eutanasia  □ Miltefosine  □ Other |
| --- | --- |

1. In case you selected “Other”, which treatment is it?

**6^th^ Clinical case**

Dog, male, 3 years old. Lives in a geographic area where leishmaniosis is not endemic but will move to an endemic zone. Therefore, came for screening for leishmaniosis and, then, apply prophylaxis.

**General examination:** normal, without abnormalities.

1. Which prophylaxis would you do? Select one or more options.

□ Domperidone □ Repellent/insecticide □ Vaccination

**THIRD PART – OTHER QUESTIONS**

This third and last part refers to the current guidelines concerning diagnosis and treatment of canine leishmaniosis.

1. Do you know about any guidelines for diagnosis and treatment of leishmaniosis?

□ No □ Yes

1. If yes, which ones do you prefer to use?

| □ World Health Organization (WHO)  □ European Food Safety Authority (EFSA)  □ European Scientific Counsel Companion Animal Parasites (ESCCAP)  □ World Organization for Animal Health (OIE) | □ Canine Leishmaniosis Working  Group (CLWG)  □ LeishVet group  □ Other  □ I have knowledge, but I do not apply any specific guidelines. |
| --- | --- |

1. In case you selected “Other”, which one is it?

**STAGING ACCORDING WITH THE LEISHVET GUIDELINES**

Bearing in mind the LeishVet guidelines, in which stage would you include each of the following clinical cases?

| 1. 1st clinical case   □ Stage I  □ Stage II  □ Stage III  □ Stage IV  □ I prefer not to answer/I do not know | 1. 2nd clinical case   □ Stage I  □ Stage II  □ Stage III  □ Stage IV  □ I prefer not to answer/I do not know | 1. 3rd clinical case□ Stage I   □ Stage II  □ Stage III  □ Stage IV  □ I prefer not to answer/I do not know |
| --- | --- | --- |
| 1. 4th clinical case   □ Stage I  □ Stage II  □ Stage III  □ Stage IV  □ I prefer not to answer/I do not know | 1. 5th clinical case   □ Stage I  □ Stage II  □ Stage III  □ Stage IV  □ I prefer not to answer/I do not know |  |

**COMPLEMENTARY DIAGNOSTIC METHODS. TREATMENT.**

1. Besides the observation of clinical signs, which methods do you use to diagnose leishmaniosis in a routine basis? Select one or more options.

□ Cytology □ PCR

□ Histopathology □Serology – ELISA

□ Immunohistochemistry □Serology – IFAT

□ Other □Serology – immunochromatography (rapid test)

1. In case you selected “Other”, which one is it?
2. In case you use PCR, which samples do you prefer to use? Select one or more options.

| □ Spleen  □ Buffy coat  □ Lymph nodes  □ Bone marrow  □ I do not use PCR | □ Other  □ Skin  □ Blood  □ Urine  □ Conjunctival swabs |
| --- | --- |

1. In case you selected “Other”, which one is it?
